# Cancer specialist nurses’ perspectives of physical activity promotion and the potential role of physical activity apps in cancer care

**DOI:** 10.1007/s11764-019-00801-w

**Published:** 2019-09-02

**Authors:** Anna L. Roberts, Henry W. W. Potts, Claire Stevens, Phillippa Lally, Lee Smith, Abigail Fisher

**Affiliations:** 1 Department of Behavioural Science & Health, University College London, Gower Street, London, WC1E 6BT UK; 2 Institute of Health Informatics, University College London, London, UK; 3 The Cambridge Centre for Sport and Exercise Sciences, Department of Life Sciences, Anglia Ruskin University, Cambridge, UK

**Keywords:** Physical activity, Nurse, Oncology, Cancer survivorship, Smartphone app, Intervention

## Abstract

**Purpose:**

The purpose of this study was to understand breast, prostate and colorectal cancer clinical nurse specialists’ (CNSs) perspectives on physical activity (PA) promotion and the role of smartphone apps to support PA promotion in cancer care.

**Methods:**

CNSs working in breast, prostate or colorectal cancer were recruited via advertisements distributed by professional organizations. In-depth semi-structured telephone interviews were conducted and analysed using thematic analysis.

**Results:**

Nineteen CNSs participated. The analysis resulted in 4 themes regarding CNSs’ perspectives of PA promotion within cancer care: (i) policy changes in survivorship care have influenced CNSs’ promotion of PA; (ii) CNSs recognize their role in supporting PA but sit within a wider system necessary for effective PA promotion; (iii) CNSs use several techniques to promote PA within their consultations; (iv) remaining challenges in PA promotion. The analysis resulted in 3 themes regarding CNSs’ perspectives on the use of apps to promote PA within cancer care: (i) the influence of apps on access to PA support; (ii) the role of apps in self-directed PA; (iii) implementing apps in cancer care.

**Conclusions:**

The results of this study provide valuable insight into the CNS role and provide a number of important considerations for the development and implementation of PA interventions within cancer care, with a specific focus on smartphone-based interventions.

**Implications for Cancer Survivors:**

CNSs play an important role in PA promotion in cancer care and this research can inform the development of PA interventions delivered via smartphone app for people affected by cancer.

## Introduction

Over 14 million people worldwide are diagnosed with cancer each year, and this is expected to rise to 22 million over the next two decades [1]. In the United Kingdom (UK), it is estimated that 1 in 2 people born after 1960 will be diagnosed with cancer at some point in their lifetime [2]. Despite increasing cancer incidence, the number of people living with and beyond the disease is also increasing. In 2012, there were 32 million people worldwide living beyond 5 years of diagnosis [1]. In 2015, it was estimated that there were over 2.5 million people living after a diagnosis of cancer in the UK, and this is expected to increase by 3% each year, to reach almost 4 million by 2030 [3].

There are many common physical and psychological consequences of cancer and treatment that can have a profound, and often long-term impact on the quality of life and wellbeing of people living with/beyond cancer (LWBC), including fatigue [4, 5], pain [6], sleep disturbance [7], lymphoedema [8, 9], weight gain [10, 11], loss of muscle mass [12], cancer-related distress (e.g. fear of recurrence, financial concerns) [13], anxiety [14] and depression [15]. As a result, people LWBC report worse health-related quality of life (HRQoL) when compared with the general population [16]. Furthermore, the majority of people LWBC are living with at least one other long-term chronic condition (e.g. hypertension, obesity, mental health conditions) [17, 18]. The shared risk factors between cancer and heart disease, and toxicity of cancer treatment, can also leave people LWBC at increased risk of cardiovascular disease [19, 20].

### The shift in survivorship care in the UK

As the number of people LWBC increases, there has been growing recognition of the need to provide support to prevent and/or manage the physical, psychological, social, financial and information issues faced by people LWBC. The National Cancer Survivorship Initiative (NCSI) was launched in the UK in 2007, which involved a collaboration between the UK government’s Department of Health, the National Health Service (NHS) and the national cancer charity, Macmillan Cancer Support [21]. The NCSI made several recommendations to better support the needs of people LWBC and integrate survivorship care. One of the key products that was developed and tested by the NCSI collaboration was the “Recovery Package”. The Recovery Package includes a holistic needs assessment (HNA), treatment summary, cancer care review and a health and wellbeing event. The HNA is a questionnaire that highlights a person’s most important physical, practical, emotional, spiritual and social needs or concerns and can be used by their healthcare professionals (HCPs) in appropriate treatment and care planning. The four components of the Recovery Package aim to provide a support and self-management package focused on the promotion of physical activity (PA) as part of a healthy lifestyle, more effective management of the consequences of cancer treatment and greater provision of information, financial and work support [22]. The Independent Cancer Taskforce recommended that everyone diagnosed with cancer in the UK should have access to the elements of the Recovery Package, including advice on healthy lifestyle and PA by 2020 [23]. This was upheld in the recently published NHS Long Term Plan [24]. PA, in particular, is highlighted in the Recovery Package due to evidence from randomized controlled trials (RCTs) of PA interventions that show improvements in fatigue [25–27], pain [25, 28], sleep [25, 26, 28], lymphoedema [29, 30], anxiety and depression [25, 26, 31], body composition [32, 33] and quality of life [25, 26, 34] in people LWBC. Furthermore, there is a large body of observational evidence that shows that people LWBC who are more active have reduced all-cause and cancer-specific mortality risk and a reduced risk of cancer recurrence [35–42].

### Challenges in delivering PA advice among healthcare professionals

People LWBC are keen to receive PA and lifestyle advice in the context of cancer from their clinical team, but report feeling the amount of guidance they had received was unsatisfactory [43, 44]. Only 31% of 15,254 peopled diagnosed with colorectal cancer in the UK recalled having received any PA advice as part of their cancer care, but those who recalled receiving advice were more likely to meet PA guidelines [45]. A RCT demonstrated that an oncologists’ recommendation to increase PA led to a significant increase in self-reported PA versus usual care, in women diagnosed with breast cancer [46].

However, a survey of 460 nurses, surgeons, physicians and allied health professionals caring for cancer patients in the UK revealed that 36% were unaware of any lifestyle guidelines and 51% were unaware of PA guidelines for cancer patients [47]. Healthcare professionals’ (HCPs) barriers to providing lifestyle advice to patients included the patient being too frail or unwell (70%), perceived lack of patient interest (48%), lack of time (36%), not being the right person to provide advice (25%) and lack of clear guidelines (25%) [47]. Qualitative interviews with 21 HCPs from this study revealed further concerns about a potential loss of connection with the patient and fear of the patient feeling blamed or guilty as a result of provision of lifestyle advice, particularly for patients who live in areas of high deprivation or who may face other socioeconomic barriers to improving their health behaviours [48]. A small survey of 48 HCPs involved in cancer care in Ireland revealed that 86% acknowledged the value of PA among people LWBC and that 88% agreed that discussing PA was part of their role [49]. However, when asked to provide examples of PA recommendations provided, 42% did not provide advice that aligned with current PA guidelines for people LWBC and 12% did not provide any PA advice.

### Nurses’ perceptions of providing PA advice in cancer care

Nurses have been identified as a critical HCP group for the delivery of nutrition, diet and lifestyle advice among people LWBC [50]. Almost half (46%) of 327 Dutch oncology nurses felt they had insufficient knowledge to provide advice on PA [51]. A survey of 274 oncology nurses in the USA found that 75% of nurses reported enquiring about PA and approximately two-thirds gave PA recommendations [52]. A study of 119 oncology nurses in Australia and New Zealand revealed that they perceived themselves to be the major providers of PA advice to their patients and promoted PA before, during and after treatment [53]. The nurses in these studies reported several perceived benefits of PA among their patients including improvements in quality of life, mental health, coping, ability to carry out activities of daily living and attenuating physical declines from treatment. However, barriers to PA promotion included lack of time, lack of adequate support structures, perceived lack of interest from patients, being unsure what to recommend and potential risks to the patients [52, 53].

There has been less research on oncology nurses’ views in the UK. This is particularly important given the large variation in healthcare systems, nursing training and cancer pathways in different countries and the relatively recent change in the approach to survivorship care. With a greater and more specific focus on the promotion of PA as part of the Recovery Package, it is therefore possible that the attitudes and perceptions of HCPs with regard to provision of lifestyle and PA advice may have changed during its implementation across the UK. As the majority of the co-ordination and delivery of the elements of the Recovery Package is usually carried out or overseen by the Clinical Nurse Specialist (CNS), they play a key role in effective PA promotion in cancer care in the UK. In the UK, CNSs are senior, experienced registered nurses, who have specialist knowledge, skills and competence in their clinical field. They are often educated to at least Masters level, although this is not currently mandated.

### The potential for delivering PA interventions via smartphones in cancer care

A systematic review and meta-analysis published by our research group has shown that digital interventions (e.g. websites, smartphone apps) may increase the moderate-vigorous PA participation of people LWBC by up to 49 min per week [54]. Our previous work has also found that people diagnosed with breast, prostate and colorectal cancers are receptive to the use of smartphone apps in the promotion of PA [55]. The participants in this study also stated that recommendations to appropriate PA apps should be integrated into routine cancer care and they identified their CNS as a key HCP who could direct them to an appropriate app [55]. Furthermore, a recent survey of 611 haematology cancer patients reported that 82% would use a health app if their HCP recommended it to them [56]. The recruitment approach and physical settings (e.g. the healthcare system) have also been identified as important in engagement with digital interventions more broadly [57, 58]. The importance of seeking the views and input of those who are involved in the delivery of an intervention, as well as intended users, in the development of an intervention is well-established [59, 60]. Therefore, this study aimed to understand breast, prostate and colorectal cancer CNSs’ perspectives on PA promotion and the role of smartphone app–based PA interventions in cancer care.

## Methods

### Participant recruitment

Breast, prostate and colorectal cancer CNSs, based in the UK, were recruited via study advertisements distributed through professional organizations including the Contact, Help, Advice and Information Network, the UK Oncology Nursing Society, a Macmillan Cancer Support nursing review panel, the National Colorectal Cancer Nurses Network and via existing contacts within the NHS. An initial total recruitment target of 21 participants was set, with an aim to recruit approximately 7 nurses from each of the 3 cancer types. If new themes continued to be identified after analysis of these 21 interviews, recruitment would continue until saturation was achieved. Participants were offered a £25 gift voucher as a token of appreciation for completing the study.

### Ethical approval

Ethical approval for this study was granted by the UCL Research Ethics Committee (reference: 7663/002). Participants were informed of the study purpose and of their rights via a written information sheet and returned a signed consent form to take part in the study. Informed consent was obtained from all individual participants included in the study.

### Procedure

Participants took part in a telephone interview between January and October 2018. A semi-structured interview schedule (Table 1) was used as a guide. Interviews were audio-recorded and transcribed verbatim.

**Table 1:** Semi-structured interview guide

Discussion point	Details
Introductions	Brief introductions and confirm participant details including gender, cancer specialty and region of the UK in which they are working. Gain verbal consent to audio-record interview, check time available.
Background and current practice	Ask whether participant currently discusses PA with patients If yes, ask to describe: • when and how discussions occur • how patients respond • what is discussed/recommended • what resources/support are patients directed to If no: • Explore reasons why not and barriers
The CNS role in PA promotion	Ask about their thoughts on their role to discuss PA with their patients. Prompts: • Role of other health professional groups in PA promotion • Feedback patient preference for CNS signposting and ask to comment
PA apps in cancer care	Ask participant initial thoughts on using apps to promote PA apps among their patients. Prompts: • Opportunities and challenges faced with digital/app-based PA support • Types of PA that could be supported via apps • Types of PA apps they would recommend to their patients • Pros and cons of cancer-specific vs. generic PA apps • Factors that would encourage or discourage recommending a patient to use a PA app (e.g. patient sociodemographic or disease characteristics including cancer type, stage, prognosis, treatment) • Do they currently recommend any apps to their patients (PA-based or otherwise)?
Implementation	Ask participant to discuss how the implementation/integration of a PA app–based intervention within routine cancer care might be most successful in practice. Prompts: • What resources, training or healthcare system changes might be required? • How could other CNSs be informed? • How could it be rolled out across a region/the country?

### Analysis

Interview transcripts were analysed using an inductive, data-driven approach to the six-stage process of thematic analysis described by Braun and Clarke [61]. The initial phase of analysis began by AR reading and re-reading the first 11 interview transcripts in order to familiarize with the data, then by iteratively assigning passages of text to relevant codes. The initial codes were refined and further specified to develop an initial coding framework, which was developed by generating new codes when existing codes were not deemed appropriate. Sub-codes were created to further specify aspects of the data. This coding framework was developed in order to meet the two aims of this study (to understand breast, prostate and colorectal cancer CNSs’ perspectives of PA promotion and the role of smartphone app–based PA interventions in cancer care) which guided the analysis. The framework was revised during several rounds of data analysis on all of the interview transcripts and was used by a second researcher (CS) to code 4 (21%) of the total interviews. In collaboration with CS, a revised, final coding framework was created, with minor discrepancies agreed via discussion. No new codes were identified, saturation was reached and recruitment was concluded. The final codes were then applied to all of the interview transcripts and incorporated into appropriate themes or sub-themes during discussion. Data analysis was conducted in NVivo 12.

## Results

### Sample characteristics

Thirty CNSs expressed interest in the study, 19 returned consent forms and completed the telephone interview. Data saturation was deemed to have been met after analysis of these 19 interviews and recruitment finished. Of these 19 nurses, 18 (95%) were female, 9 (47%) were colorectal cancer CNSs, 6 (32%) were prostate cancer CNSs and 4 (21%) were breast cancer CNSs.

### Thematic analyses

The analysis was guided by the two aims of this study. The interview discussions tended to focus less so on the role of smartphone app–based PA interventions and more so on PA promotion more broadly, and this is reflected in these results.

### CNSs’ perspectives of physical activity promotion within cancer care

Regarding CNSs’ perspectives of PA promotion within cancer care, the analysis resulted in 4 key themes: (i) policy changes in survivorship care have influenced CNSs’ promotion of PA; (ii) CNSs recognize their role in supporting PA but sit within a wider system necessary for effective PA promotion; (iii) CNSs use several techniques to promote PA within their consultations; (iv) remaining challenges in PA promotion.

#### Policy changes in cancer survivorship care have influenced CNSs’ promotion of physical activity

Many of the CNSs discussed the impact that the shift in focus in survivorship care and the “Living With and Beyond Cancer Initiative” has had on cancer care and PA promotion in their role:

it’s very much about the survivorship package and…trying to engage with, you know, the Department of Health Cancer Strategy…so our cancer lead-matron, with the rest of the team, has engaged with a program of, you know, Living With and Beyond Cancer and survivorship. And PA is very much a part of that. And so, over the last few years, you know, um, our offering to patients of support for those types of things has increased (prostate cancer CNS)

The implementation of the Recovery Package across the UK was described as an important opportunity to facilitate a conversation about PA:

certainly all of our patients will be having Holistic Needs Assessments and diet and exercise and lifestyle in general it comes up on the Holistic Needs Assessment, so it’s a good time to just kind of mention it and that’s how I’ve picked up a couple recently through doing holistic needs and you know, looking up information for them and giving them the DVDs and stuff (colorectal cancer CNS)

#### CNSs recognize their role in supporting physical activity but sit within a wider system necessary for effective physical activity promotion

The CNSs stated that discussing and supporting PA with their patients was an accepted and key part of their role:

I definitely see that within the role of a CNS nurse…I see my role as helping people live well with their cancer…and exercise is a part of actually, erm, feeling well and good about yourself…I just think it’s part of our job (prostate cancer CNS)

CNSs explained that because patients are often under their care throughout the cancer trajectory, they are able to form relationships, develop a holistic understanding of their circumstances and how their diagnosis and treatment has affected them. Therefore, they described feeling that they are in a good position to inform patients about PA, signpost and refer to further PA support, and continue to promote and support PA throughout treatment and follow-up:

the CNS is the one that you have the relationship with for patients… the CNS is the one constant in their whole cancer pathway and relationships are forged and built very quickly with our patients...and actually, to be honest, it’s the CNS that always gets asked by the patient (prostate cancer CNS)

Some CNSs discussed the increased pressure faced by the health service and how this is affecting job roles, including the introduction of a relatively new “support worker” role. CNSs explained that support workers are being/have been introduced in many hospitals to support CNSs with tasks that can be carried out by staff without nursing qualifications (e.g. administrative tasks and signposting patients to information and support). CNSs stated that support workers could be involved in some aspects of PA promotion and in some cases this was happening already:

we are getting more and more support workers which is an excellent role…one of the domains of their job would be to be promoting PA…there’s a national shortage of nurses and particularly, erm Clinical Nurse Specialists…and when somebody retires or leaves their post in the NHS…their post is downgraded, partly to save money but also partly because there’s no one with the experience to fill it at a Band 7 role (prostate cancer CNS)

However, there were potential concerns about whether support workers delivering PA support have the same amount of credibility among patients and whether their role in PA promotion may be as influential:

many of our queries are not complex, you know, they’re, ‘When’s my scan?’, ‘When’s my appointment?’ There’s a lot of things that can be done by someone else but…what I worry about is with, erm, sort of Band 4s I suppose is that they don’t have that kind of in-depth knowledge of how things benefit, you know, or just the breadth of experience to, you know, be convincing. Would they just be like ticking boxes – ‘Oh right, I’ve talked about exercise’ and move on to the next subject sort of thing (prostate cancer CNS)

While the CNSs were very aware of their role in PA promotion, they also discussed the importance of other HCPs in delivering PA recommendations, advice and support for more effective PA promotion:

I do think it probably is part of our role to be doing that but I don’t think it’s solely our role…if they’re seeing physio, for example, when they’ve had surgery, then there’s an opportunity there to be talking to them about further exercises…we don’t always get to clinics to see patients for a follow-up, so consultants have to…take some of that responsibility as well…and…it can be an ongoing thing, so therefore GPs [General Practitioners] probably should take some ownership of it (colorectal cancer CNS)

Nurses spoke about their perception that when PA was endorsed by an oncologist or surgeon, there was a positive impact on PA promotion among patients:

“our oncologist…was very vocal about actually trying to combat, um, chemotherapy fatigue…and she was very much exercise is really positive, it really helps. She’s done loads of research into it so actually by keeping active, the reoccurrence of cancer can be reduced. So by saying stuff like that, which she was really pro-exercise in our consultations, it did encourage a lot more [patients]” (colorectal cancer CNS)

However, they also perceived that other HCPs may be less likely to promote PA among their patients:

I don’t think the message is out there yet…with some of…[the] other disciplines… interestingly surgery because you’d think that people in that field would be wanting to make sure the patients they’re operating on have got the best chance of recovering because it looks good for your personal stats…I don’t think people are aware of the…proper evidence behind actually this person’s gonna spend a day and a half less in hospital if they’re in better shape when they come. So it’s not belittled but I don’t think the value of the intervention is fully understood by all of our colleagues (colorectal cancer CNS)

Community-based exercise referral schemes for cancer survivors were described positively, among nurses who reported having the ability to refer patients to such services:

it’s cofunded by [the] CCG [clinical commissioning group] and Macmillan…they run PA classes at our Macmillan centre…but they’ll go out and do a PA assessment in someone’s home, in their local park, in their local gym, they’ll write a programme for somebody and then they can carry that on with their local gym provider or joining health walks or whatever suits them…that’s why we’re really blessed in this area to have a service where we can say, ‘Just go and see these experts’ (colorectal cancer CNS)

#### CNSs use several techniques to promote physical activity within their consultations

CNSs described several ways in which they try to promote PA to their patients, including explaining the cancer-specific benefits of PA, adapting their use of language to promote PA, promoting PA as a way to emphasize patient control and tailoring their approach to PA discussions based on individual patients.

With regards to explaining the cancer specific benefits of PA, the  CNSs described specifically recommending PA to patients as an effective way to cope with and recover from treatment, manage side effects, improve wellbeing, and reduce future cancer risk and the risk of other comorbid conditions:

when people are on hormones it’s easy to encourage [PA]. Because of all the side effects of hormones, exercise is so good to help with, with all of them and I say to everyone, you know, ‘You’ll feel tired and the best thing to do is go for a walk. Don’t lay down, don’t feel tempted.’…And especially with all, you know, weight gain and hot flushes and all the side effects really (prostate cancer CNS)

Some nurses specifically mentioned that research evidence demonstrating the benefits of PA after cancer can reinforce their recommendations and provide credibility to their advice:

I was just having a conversation with somebody this morning… we said to her, ‘Well, how much exercise are you doing?’, ‘Well, none. Erm going out maybe once every two weeks.’ ‘Well, actually the evidence suggests that the more exercise you do, the less weak you will be, the less fatigued you will feel. So actually you need to be going out more, regularly. Even if it’s just for a little walk for 10, 15 minutes down the road’ (colorectal cancer CNS)

Several nurses also described how they had changed their use of language or used a specific style of communication (e.g. motivational interviewing techniques) to promote PA:

there’s a difference between whether you signpost somebody or whether you make a referral…I used to be very much about signposting but now I’m very much about referring…I’ll say…‘I’m not prescribing any medications to you, but I’m actually prescribing PA.’ So I do tailor it, and use language (colorectal cancer CNS)

most of us have been trained now on motivational interviewing …it is a different way of talking isn’t it?...we’ve got some ideas about what’s going to be beneficial to you, but we also need to understand where you’re coming from and what the barriers and difficulties may be in taking that advice and how we can sort of maybe swing that balance by thinking about what it needs to look like, what the message needs to be for you (colorectal cancer CNS)

Emphasizing that PA is a behaviour that the patient can take control of, involve them in their care and empower patients to promote self-management was also discussed as a way to promote PA:

*I* say, ‘Look, we have done our bit, now it’s your turn to do your bit,’ you know? And so this is my mantra [laughs] you know, just after the cancer treatment and sometimes people laugh at it but…for me it works, for them it works (colorectal cancer CNS)

CNSs also described a process of adapting their conversations about PA to each individual patient, taking into account various characteristics and factors, in order to tailor their conversations, recommendations and advice. This included tailoring the conversation based on the participants’ age, baseline PA/fitness levels and comorbidities:

it depends on the age of the patient and any comorbidities that they have, obviously, because for some patients, just actually getting off the sofa and going for a ten-minute walk is the most they’ve ever done for years. Erm, so we kind of have to gauge it individually on each patient…it’s down to pre-existing conditions and things like that (prostate cancer CNS)

Conversations about PA were also tailored based on each patient’s experience of cancer, treatment, side effects and disease stage:

it varies from patient to patient, obviously, depending on sort of what their, erm, disease state is, erm, where they are in their treatment, erm, you know, so if you’ve got somebody who is newly diagnosed it might not be appropriate or if you’ve got somebody who’s sort of near the end of life, again it might not be appropriate. So it depends. There’s no kind of one answer that fits all (colorectal cancer CNS)

Tailoring was also based on their perception of the patients’ openness to discuss PA, or how interested they seem to engage with different types of PA:

I will just take a lead from the patient to be honest, so if somebody says they’re fine and they’re back to normal, you know, I won’t necessarily sort of push the exercise agenda, you know, I’ll just say, ‘Fine, these are the things that you need to keep an eye out for, keep as active as you can, you know.’ So we do try, and sort of discuss it with everyone (breast cancer CNS)

I think it’s down to the individual to say, you know, what they want to do… there will be the person who just wants to read the information, to see, well, maybe they want to just move around a little bit more at home. And there are those that will go out and join a walking group…there are those that will engage with the gym. Um, there are those that are going to get on the bike and do some exercise at home. There’s such a wide variety (prostate cancer CNS)

#### Remaining challenges in physical activity promotion

Among some CNSs interviewed, there was a lack of accurate knowledge of the PA guidelines and how best to provide advice to patients. In some instances, this led to reports of recommendations provided that were inaccurate, somewhat vague or at odds with current PA guidelines for cancer survivors:

[we recommend] not to exercise up to the lead up to surgery, but after they’ve had surgery…we don’t say don’t exercise at all, we say do gentle exercise and increase it slowly over time…if they go to the gym regularly and work out, then we would say then don’t do that type of thing (colorectal cancer CNS)

I probably should know [the guidelines] but I don’t…it’s just common sense, I think, because we’ve been doing this for so long, erm, it’s sort of second nature…We talk about, you know, doing their, the ten-minute walk a day, you know, simple guidelines from the government…we probably cover it not realising we’re covering the guidelines (breast cancer CNS)

The CNSs also described difficulty with understanding of the strength and resistance training element of the guidelines and how to communicate that with patients:

“I think people don’t know what to do for [strength and resistance-based training]…we know with hormonal therapy, people lose their muscle tone and some of the men are quite distressed by that, but it’s a bit difficult…I think it’s very difficult to find, erm, clear guidelines on what that actually means and how they can do it without incurring too much expense...we tend not to say anything because we don’t know what we’re meant to be recommending” (prostate cancer CNS)

CNSs also reported particular concerns about the potential safety of the recommendations they provide and therefore reported feeling that the advice they provide may be overly cautious:

it’s a bit like the blind leading the blind a bit sometimes because they come to us and ask us can they do specific things but everyone always errs on the side of caution because we don’t want to cause more harm than good but then at the back of your mind you’re thinking, you know, it’s what they’re used to, it’s what they want to do and it’s, you know, psychologically, it’s going to be beneficial for them (breast cancer CNS)

Some nurses described the need to involve other healthcare or exercise professionals to provide the specialist knowledge/input that they felt unable to advise on:

what I would say to them is if they wanted to go to a gym or something…I mean, you know, there’s no reason why they shouldn’t eventually, but, erm, I think I would say to them, you know, talk to your GP or talk to your consultant or talk to somebody in a gym for advice as to what you should and you shouldn’t be doing (colorectal cancer CNS)

However, they acknowledged that they can often only refer particularly complex patients to Physiotherapists and/or Occupational Therapists, but the majority of inactive patients may not meet the need for this type of referral. Therefore, the majority of inactive patients may lack specific support to increase PA:

it’s the tricky bit…the ones who…do exercise, you, they carry on, lovely, the ones who can’t, you know, there’s not a lot you can do if someone cannot do any exercise, but then it may be obvious – you know, using physio, or OT [Occupational Therapy] to encourage what they can do, and then it’s these ones in the middle. So it’s a way of finding out what their, what they would engage in really and I guess that’s the tricky bit (prostate cancer CNS)

The nurses also described their perception that their recommendations, advice or signposting may not be sufficient for behaviour change among some of their patients:

they basically like say, ‘Yes, OK’ [laughing] but I don’t think they actually do it, a lot of them (colorectal cancer CNS)

there’s always gonna be the minority of patients, no matter what you tell them, they’re not gonna listen and they’re gonna carry on drinking their 40 pints a week, or, you know, they’re gonna sit on their sofa 24/7. No matter how much you try and guide them in the right direction, it’s that thing, isn’t it, you can lead a horse to water but you can’t make it drink (prostate cancer CNS)

Furthermore, the nurses reported that despite the availability of appropriate, local PA services, patient barriers to attending these services may result in low uptake:

there’s like exercise groups for four men at a time and they can do like a four to six-week programme…so the access is there and we do talk to people about it, erm, whether or not they take it up or not is another challenge to get over (prostate cancer CNS)

it’s alright bringing up this about exercising, but how they’re going to get there, what’s the cost of it, err, I live on my own, you know, all these sorts of barriers that are put up. We’re sort of raising it and then at the end of the consultation we’re saying, ‘Well that, in an ideal world, that’s what we want you to do’ (breast cancer CNS)

The amount of nurse time and resources available to them for effective PA promotion were also described as challenging among some of the CNSs:

for me, it’s probably more of a time issue. I don’t have, you know, I’m here on my own so I don’t really have enough time to spend with them, to sit down and have those full conversations (colorectal cancer CNS)

it is in terms of resources. We’re already down a breast care nurse and…we’re coming to that age group where a lot of nurse specialists are hitting into their 50s and so quite a lot are taking retirement around 55. So we’ve got two semi-retirements within our service here and they’re not replacing at the minute (breast cancer CNS)

### CNSs’ perspectives about the use of apps for physical activity promotion within cancer care

Regarding CNSs’ perspectives on the use of apps to promote PA within cancer care, the analysis resulted in 3 key themes: (i) the influence of apps on access to PA support; (ii) the role of apps in self-directed PA; (iii) implementing apps in cancer care.

#### The influence of apps on access to PA support

The nurses identified that one of the main advantages of app-based PA support is the potential increase in accessibility to a PA intervention as a result of the high ownership of smartphones. However, this was also discussed with the caveat that not all of their patients used smartphones or had the digital skills/confidence to engage with an app-based intervention:

I think that’s a really good idea. I don’t know what percentage of the people have a smartphone but I’d imagine quite a majority probably have a phone where they could use that sort of format…I don’t think there are any age barriers at all to this…but have they got a phone where they can download them and have they got the IT [information technology] skills and wherewithal to navigate apps? So, um, I’m thinking there will be a section in the population who just won’t be able to access them (colorectal cancer CNS)

Apps also have the potential to alleviate some of the barriers that arise from attending face-to-face services or interventions, and it may be more convenient to engage in PA independently, supported by an app:

I think quite a lot of people’s initial impressions of leisure centres are that people go there and they’re really fit and actually, if you’ve been poorly and you’ve got a stoma, you’ve got massive body image [issues] or you’ve lost your hair through chemotherapy and stuff like that. I think actually for those patients, being able to access something in your own home to build your own confidence up gradually, is much better (colorectal cancer CNS)

CNSs stated that they felt that apps offer a different approach to delivery of a PA intervention, which therefore provides patients with increased choice about how to engage with support to increase activity:

so it would be, ‘There’s lots of choices available to you, we’ve got a physical activities team, you can see somebody face-to-face, you can have a one-off assessment, you can do a range of classes, we can link you in with an app that’s been specifically designed for people affected by cancer.’ You know, whatever the, the selling points might be. So it, it would just be adding in choice as part of the script…and it’s a group of patients for whom life choices have been significantly changed. And to be able to give that choice is very positive (colorectal cancer CNS)

#### The role of apps in self-directed physical activity

Apps were also perceived to be a useful tool to help to promote self-management and patients’ ownership over their health and wellbeing:

I think it’s quite nice and I think when you say to patients we would like you to use this, you know, we want you to track this, I think they do like taking a bit of ownership for things (prostate cancer CNS)

However, CNSs did raise some potential concerns or disadvantages of app-based PA interventions, including the lack of supervision from trained exercise professionals:

there’s no one for all solution here, unless you’ve got an app that takes into account the patient’s age, the patient’s baseline fitness level and then offers a solution, erm, based on that…that takes that into account…otherwise I think you could potentially, you know, if you’re pushing your older patients too hard, you potentially have the opposite effect of what you want can’t you? (prostate cancer CNS)

The potential for a lack of continued engagement with a PA app and therefore a lack of a sustained effect on behaviour change was identified as a possible disadvantage:

I think they are a useful tool. I don’t know how useful they are on a long-term basis…I don’t know…whether it’s something that people use for like a week or two and then get a bit bored…it probably wouldn’t have done enough…you need people to use it for a good, you know, few months to get into the habit of taking regular exercise (prostate cancer CNS)

#### Implementing PA apps in cancer care

With regards to how a PA intervention could be effectively implemented in routine cancer care, CNSs said that they would need to be provided with accompanying resources to promote the app, including instructions on how to download it and discussed ways to potentially maximize the success of implementation:

I wouldn’t have any problem…saying ‘You know, this is an app worth trying,’ but I’d …[need] a little sheet to give them, saying ‘This is how you download it, this is what we recommend you do with it.’ So it’s something that you can give them with their physical activities thing, with their physical activities information you’re already giving them (colorectal cancer CNS)

CNSs also reported being willing to promote PA apps provided there was an evidence-base underpinning their use, or if a particular app had been recommended for their patients by a professional organization:

very much like how web-based information now that we use, obviously we kind of signpost our patients to. And so, if there were apps developed that, you know, reflect Department of Health guidelines and things like that then obviously we’d speak to people about that and encourage people to use it (prostate cancer CNS)

I would like to use [apps] but I don’t know which ones to tell people to use and which would be the most beneficial for them really…which one’s the most effective (colorectal cancer CNS)

However, they said they would need very clear guidelines about who they could recommend an app to, in order to avoid potential blame if a patient was to injure themselves while using an app:

I’d have no problem with [recommending an app] actually if there was a guideline to say, you know, a patient whose performance status is like zero, and the, you know their fitness is at this level, then this is their target…because I’m not a fitness expert at all…if you’re recommending something it needs to be, it needs to be in black and white and authorised really to be saying those things, because if they go and pull a muscle, they do something detrimental then you don’t want to be sued, do you? (colorectal cancer CNS)

There were mixed opinions with regards to whether the CNSs thought patients would prefer publicly available PA apps that can be downloaded from commercial app stores versus an app that has specifically been developed for people affected by cancer:

Do patients that have cancer want something specific? Probably, they do, I should imagine, because they have gone through a different experience, you know? And their specifics or their requirements might be related to the consequences of treatment…because if you’ve had cancer you’d probably want something specific to that, you know, from an understanding point of view of people’s capabilities and abilities (prostate cancer CNS)

I think if there’s something already made and it works there’s no point, err, making it different. And also I think if you had something that was just for cancer, it’s permanently reminding people that they’ve got cancer or they’ve had cancer, whereas if you just did it as a general app…that helps to normalise things*.* (prostate cancer CNS)

## Discussion

The purpose of this study was to understand breast, prostate and colorectal cancer CNSs’ perspectives on PA promotion and the role of smartphone app–based PA interventions in cancer care.

### CNSs’ perspectives on PA promotion in cancer care

The CNSs in this study described their perception that PA promotion has increased as a result of the greater focus on survivorship and the LWBC Initiative within cancer care. This has led to increased opportunities to discuss PA (e.g. via the interventions included as part of the Recovery Package) and as a result, PA promotion is regarded as a key and accepted part of the CNS role. The nurses also described their increased knowledge about the benefits of PA after a cancer diagnosis (e.g. improving coping with, and recovering from treatment, managing side effects, improving wellbeing and the potential to reduce future cancer risk and risk of other common comorbid conditions). The identification of these benefits as facilitators to discussions about PA among cancer patients has also been reported in previous surveys of oncology nurses working in the USA, Australia and New Zealand [52, 53]. Discussing these benefits with patients and providing education as to how PA can improve many of the side effects or challenges faced by patients was interpreted as a specific technique to encourage patients to increase PA. Other nurses mentioned how they had made a conscious change to their language or communication style during consultations regarding PA. Examples included nurses specifically saying that they were “prescribing” PA or “referring” to services, as opposed to “signposting” and using techniques such as motivational interviewing. Motivational interviewing has been highlighted as having the potential for nurses to help patients improve their lifestyles [62]. The nurses also said that they felt that it was important to emphasize PA as a part of cancer treatment and care that the patient can take control over, to empower them to play an active role in their health and wellbeing, and promote self-management. This has also been echoed by patients in our previous work [55]. Emphasizing this aspect of control may be an effective way to promote PA among cancer patients.

Some CNSs described examples of PA recommendations provided to patients that were inaccurate, vague or at odds with established PA guidelines for cancer survivors, and the strength-resistance training element of the recommendations was described as being particularly difficult to communicate with patients. Previous research has suggested that 42% of HCPs involved in cancer care (including nurses) did not provide advice that aligned with current PA guidelines for cancer survivors [49]. Therefore, improving nurses’ knowledge and understanding of the PA guidelines and ability to communicate them with patients is required. The CNSs were also concerned about the potential safety and risks to the patient with regard to PA promotion and similar concerns have been raised in other studies of oncology nurses [52, 53]. The nurses in this study described seeking approval from other HCPs (e.g. consultants, surgeons, allied health professionals) before advising a patient to increase PA. However, previous research has also shown limited knowledge and understanding about PA guidelines among cancer survivors in other HCP groups too [47–49]. Effective PA promotion requires a consistent message about the importance of PA after cancer from a range of HCPs involved in the patients’ care. However, the CNSs in this study described a feeling that HCPs from other disciplines might lack the knowledge of the benefits of PA for cancer, lack the skills to deliver this advice and do not perceive it to be within their job role. While the CNSs in this study agreed they were the most appropriate professional to refer patients or coordinate PA support, it is important that CNSs are not regarded as a substitute for exercise professionals, as they do not necessarily have the knowledge, skills or confidence for exercise prescription on an individual level.

The nurses in this study also discussed how they try to tailor their consultations around PA with regard to several factors about each patient including age, baseline PA levels, comorbid conditions and cancer experience (e.g. treatment, side effects, disease stage, distress) as well as the types of PA the patient is interested in, and how open or willing the patient appears to be to discussing PA. However, they described feeling that even though they take those factors into account, it can be very challenging to incorporate them into a specific recommendation for an individual patient and that the advice they give can therefore feel somewhat generic. This has been recognized in an editorial in The Lancet Oncology, which states that “it would be naïve to think that there can, or should, be a one-size-fits-all approach to suit all patients” with regard to cancer-specific PA guidelines [63]. Related to this, Santa Mina and colleagues state that the existing “guidelines are unable to advise clinicians and qualified exercise professionals about how to identify and manage many potential exercise contraindications, especially given the high degree of heterogeneity in patient risks and comorbidities” [64]. As a result, further research is needed to produce more specific PA guidelines based on various patient factors (e.g. cancer type, stage, treatment, comorbidities, body composition) and clinicians should be provided with appropriate support and training so they have the competency and confidence to adapt their recommendations to an individual patient accordingly.

### CNSs’ perspectives on the use of apps to promote PA within cancer care

The nurses described feeling that apps were a positive way to increase accessibility with PA interventions as a result of the increasing proportion of the population who own smartphones and by removing some of the barriers associated with face-to-face interventions (e.g. travel, time and confidence). However, many nurses were mindful of the fact that PA apps would not necessarily be of interest to, or useful for, specific groups of their patients, including those who do not own smartphones, do not have the skills to use them or for more complex patients who may require more tailored, supervised interventions. As a result, the nurses reported that they felt that apps are a useful tool to be able to offer in addition to, as opposed to instead of, the current PA services they discuss with patients as they can increase patient choice and flexibility with how they want to engage in PA support based on their preferences and circumstances. The CNSs discussed how apps can promote patient self-management by supporting self-directed PA, but also raised concerns around the potential safety implications of exercising independently via an app or issues with sustained engagement with a PA app. Nurses reported being willing to recommend PA apps to their patients provided there was an evidence-base underpinning their use, or if a particular app had been recommended for use among cancer patients from a professional organization. CNSs also felt that in order to ensure effective implementation of an app-based intervention in cancer care, accompanying resources would be required as well as effective dissemination among colleagues.

### Strengths and limitations

To the best of our knowledge, this is the first study to qualitatively explore CNSs’ perspectives of PA promotion within routine cancer care in the UK and offers a rich understanding of factors that may affect the development and implementation of PA interventions delivered in this context, including those delivered via smartphone app. Nurses were recruited from a range of hospitals across England and Scotland providing insight into how PA promotion differs across hospitals and regions. Qualitative methodology provides a rich understanding of people’s experiences, thoughts and opinions, and provides greater depth to the findings of quantitative studies on the provision of lifestyle advice among cancer HCPs [47, 52, 53]. However, there were a number of limitations. The sample were self-selecting and this may have led to the recruitment of nurses who are particularly interested in and enthusiastic about the promotion of PA among their patients. This may affect the conclusions drawn from this study. While the interview schedule was designed to ask questions openly and to minimize the potential for nurses to feel that they should be discussing PA with patients, social desirability bias may have led to nurses adapting their responses to ensure that they are regarded positively. Attempts to recruit a similar number of nurses who worked within each of the 3 cancer sites were made, to ensure a range of perspectives of PA promotion could be collected. However, it was particularly difficult to recruit breast cancer CNSs and only 4 of the 19 nurses interviewed in this study worked with breast cancer patients. Therefore, breast cancer CNSs opinions of PA promotion and the use of apps for this purpose in cancer care may be under-represented and may differ in comparison to prostate and colorectal cancer CNSs.

### Implications for cancer survivors and future research

The results of this study, and our previous work with people LWBC [55], demonstrate that CNSs play an important role in PA promotion within cancer care. It is well-established that seeking the views and input from those who are involved in the delivery of an intervention, as well as intended users, is a crucial stage in intervention development [59, 60]. As a result, these findings can be used to help inform the development and implementation of PA interventions and services for people LWBC. Future research should aim to further explore the impact of involving CNSs in the delivery of PA interventions in cancer care, including those that are delivered via smartphone app, and the feasibility and acceptability of using apps to promote PA in people LWBC.

## Conclusion

The aims of the present study were to explore breast, prostate and colorectal cancer CNSs’ perspectives on PA promotion and the role of smartphone app–based PA interventions in cancer care. The analysis resulted in 4 themes regarding CNSs’ perspectives of PA promotion within cancer care: (i) policy changes in survivorship care have influenced CNSs’ promotion of PA; (ii) CNSs recognize their role in supporting PA but sit within a wider system necessary for effective PA promotion; iii) CNSs use several techniques to promote PA within their consultations; (iv) remaining challenges in PA promotion. There were 3 key themes regarding CNSs’ perspectives on the use of apps to promote PA within cancer care: (i) the influence of apps on access to PA support; (ii) the role of apps in self-directed PA; (iii) implementing apps in cancer care. The results of this study provide valuable insight into the CNS role and reveals a number of important considerations for the development and implementation of PA interventions within cancer care, with a specific focus on smartphone-based interventions.

## References

[1] Ferlay J, Soerjomataram I, Dikshit R, Eser S, Mathers C, Rebelo M, Parkin DM, Forman D, Bray F (2015). Cancer incidence and mortality worldwide: sources, methods and major patterns in GLOBOCAN 2012. Int J Cancer.

[2] Ahmad AS, Ormiston-Smith N, Sasieni PD (2015). Trends in the lifetime risk of developing cancer in Great Britain: comparison of risk for those born from 1930 to 1960. Br J Cancer.

[3] Maddams J, Utley M, Moller H (2012). Projections of cancer prevalence in the United Kingdom, 2010-2040. Br J Cancer.

[4] Weis J (2011). Cancer-related fatigue: prevalence, assessment and treatment strategies. Expert Review of Pharmacoeconomics & Outcomes Research.

[5] Husson O, Mols F, van de Poll-Franse L, de Vries J, Schep G, Thong MSY (2015). Variation in fatigue among 6011 (long-term) cancer survivors and a normative population: a study from the population-based PROFILES registry. Support Care Cancer.

[6] van den Beuken-van Everdingen MH (2016). Update on prevalence of pain in patients with cancer: systematic review and meta-analysis. J Pain Symptom Manag.

[7] Otte JL, Carpenter JS, Manchanda S, Rand KL, Skaar TC, Weaver M, Chernyak Y, Zhong X, Igega C, Landis C (2015). Systematic review of sleep disorders in cancer patients: can the prevalence of sleep disorders be ascertained?. Cancer Medicine.

[8] Hayes SC, Janda M, Cornish B, Battistutta D, Newman B (2008). Lymphedema after breast cancer: incidence, risk factors, and effect on upper body function. J Clin Oncol.

[9] Norman SA, Localio AR, Potashnik SL, Simoes Torpey HA, Kallan MJ, Weber AL, Miller LT, DeMichele A, Solin LJ (2009). Lymphedema in breast cancer survivors: incidence, degree, time course, treatment, and symptoms. J Clin Oncol.

[10] Winkels RM, Snetselaar T, Adriaans A, van Warmerdam LJC, Vreugdenhil A, Slooter GD, Straathof JW, Kampman E, van Lieshout R, Beijer S (2016). Changes in body weight in patients with colorectal cancer treated with surgery and adjuvant chemotherapy: an observational study. Cancer Treatment and Research Communications.

[11] Newton RU, Jeffery E, Galvão DA, Peddle-McIntyre CJ, Spry N, Joseph D, Denham JW, Taaffe DR (2018). Body composition, fatigue and exercise in patients with prostate cancer undergoing androgen-deprivation therapy. BJU Int.

[12] Smith MR, Saad F, Egerdie B, Sieber PR, Tammela TLJ, Ke C, Leder BZ, Goessl C (2012). Sarcopenia during androgen-deprivation therapy for prostate cancer. J Clin Oncol.

[13] Beckjord EB, Reynolds KA, van Londen GJ, Burns R, Singh R, Arvey SR, Nutt SA, Rechis R (2014). Population-level trends in posttreatment cancer survivors’ concerns and associated receipt of care: results from the 2006 and 2010 LIVESTRONG surveys. J Psychosoc Oncol.

[14] Mitchell AJ, Ferguson DW, Gill J, Paul J, Symonds P (2013). Depression and anxiety in long-term cancer survivors compared with spouses and healthy controls: a systematic review and meta-analysis. Lancet Oncol.

[15] Krebber AM (2014). Prevalence of depression in cancer patients: a meta-analysis of diagnostic interviews and self-report instruments. Psychooncology.

[16] Quinten C, Coens C, Ghislain I, Zikos E, Sprangers MA, Ringash J, Martinelli F, Ediebah DE, Maringwa J, Reeve BB, Greimel E, King MT, Bjordal K, Flechtner HH, Schmucker-von Koch J, Taphoorn MJ, Weis J, Wildiers H, Velikova G, Bottomley A, PROBE, EORTC Clinical Groups (2015). The effects of age on health-related quality of life in cancer populations: a pooled analysis of randomized controlled trials using the European Organisation for Research and Treatment of Cancer (EORTC) QLQ-C30 involving 6024 cancer patients. Eur J Cancer.

[17] Macmillan Cancer Support. *The burden of cancer and other long-term health conditions*. 2015; Available from: https://www.macmillan.org.uk/documents/press/cancerandotherlong-termconditions.pdf. Accessed 24 Apr 2019.

[18] Cummings A, Grimmett C, Calman L, Patel M, Permyakova NV, Winter J, Corner J, Din A, Fenlon D, Richardson A, Smith PW, Foster C, Members of CREW Study Advisory Committee (2018). Comorbidities are associated with poorer quality of life and functioning and worse symptoms in the 5 years following colorectal cancer surgery: results from the ColoREctal well-being (CREW) cohort study. Psychooncology.

[19] Hooning MJ, Botma A, Aleman BMP, Baaijens MHA, Bartelink H, Klijn JGM, Taylor CW, van Leeuwen FE (2007). Long-term risk of cardiovascular disease in 10-year survivors of breast cancer. J Natl Cancer Inst.

[20] Khan NF, Mant D, Carpenter L, Forman D, Rose PW (2011). Long-term health outcomes in a British cohort of breast, colorectal and prostate cancer survivors: a database study. Br J Cancer.

[21] Richards M, Corner J, Maher J (2011). The National Cancer Survivorship Initiative: new and emerging evidence on the ongoing needs of cancer survivors. Br J Cancer.

[22] Macmillan Cancer Support. *The Recovery Package*. Available from: https://www.macmillan.org.uk/about-us/health-professionals/programmes-and-services/recovery-package#297615. Accessed 24 Apr 2019.

[23] Independent Cancer Taskforce. Achieving world-class cancer outcomes - a strategy for England . 2015; Available from: https://www.cancerresearchuk.org/sites/default/files/achieving_world-class_cancer_outcomes_-_a_strategy_for_england_2015-2020.pdf. Accessed 24 Apr 2019.

[24] NHS England. The NHS long term plan. 2019; Available from: https://www.longtermplan.nhs.uk/wp-content/uploads/2019/01/nhs-long-term-plan.pdf. Accessed 24 Apr 2019.

[25] Mishra SI (2012). Exercise interventions on health-related quality of life for cancer survivors. Cochrane Database Syst Rev.

[26] Mishra SI (2012). Exercise interventions on health-related quality of life for people with cancer during active treatment. Cochrane Database Syst Rev.

[27] Kessels E, Husson O, van der Feltz-Cornelis CM (2018). The effect of exercise on cancer-related fatigue in cancer survivors: a systematic review and meta-analysis. Neuropsychiatr Dis Treat.

[28] Nakano J, Hashizume K, Fukushima T, Ueno K, Matsuura E, Ikio Y, Ishii S, Morishita S, Tanaka K, Kusuba Y (2018). Effects of aerobic and resistance exercises on physical symptoms in cancer patients: a meta-analysis. Integrative Cancer Therapies.

[29] Baumann FT, Reike A, Hallek M, Wiskemann J, Reimer V (2018). Does exercise have a preventive effect on secondary lymphedema in breast cancer patients following local treatment? - a systematic review. Breast Care.

[30] Baumann FT, Reike A, Reimer V, Schumann M, Hallek M, Taaffe DR, Newton RU, Galvao DA (2018). Effects of physical exercise on breast cancer-related secondary lymphedema: a systematic review. Breast Cancer Res Treat.

[31] Patsou, E.D., et al., Effects of physical activity on depressive symptoms during breast cancer survivorship: a meta-analysis of randomised control trials. Esmo Open, 2017. **2**(5). 10.1136/esmoopen-2017-000271 PMC5729305 29259819

[32] Soares Falcetta F, de Araújo Vianna Träsel H, de Almeida FK, Rangel Ribeiro Falcetta M, Falavigna M, Dornelles Rosa D (2018). Effects of physical exercise after treatment of early breast cancer: systematic review and meta-analysis. Breast Cancer Res Treat.

[33] Dieli-Conwright CM, Courneya KS, Demark-Wahnefried W, Sami N, Lee K, Buchanan TA, Spicer DV, Tripathy D, Bernstein L, Mortimer JE (2018). Effects of aerobic and resistance exercise on metabolic syndrome, sarcopenic obesity, and circulating biomarkers in overweight or obese survivors of breast Cancer: a randomized controlled trial. J Clin Oncol.

[34] Zhang X, Li Y, Liu D (2019). Effects of exercise on the quality of life in breast cancer patients: a systematic review of randomized controlled trials. Support Care Cancer.

[35] Lahart IM, Metsios GS, Nevill AM, Carmichael AR (2015). Physical activity, risk of death and recurrence in breast cancer survivors: a systematic review and meta-analysis of epidemiological studies. Acta Oncol.

[36] Schmid D, Leitzmann MF (2014). Association between physical activity and mortality among breast cancer and colorectal cancer survivors: a systematic review and meta-analysis. Ann Oncol.

[37] Wu WR (2016). Pre- and post-diagnosis physical activity is associated with survival benefits of colorectal cancer patients: a systematic review and meta-analysis. Oncotarget.

[38] Meyerhardt JA, Heseltine D, Niedzwiecki D, Hollis D, Saltz LB, Mayer RJ, Thomas J, Nelson H, Whittom R, Hantel A, Schilsky RL, Fuchs CS (2006). Impact of physical activity on cancer recurrence and survival in patients with stage III colon cancer: Findings from CALGB 89803. J Clin Oncol.

[39] Li T, Wei S, Shi Y, Pang S, Qin Q, Yin J, Deng Y, Chen Q, Wei S, Nie S, Liu L (2016). The dose-response effect of physical activity on cancer mortality: findings from 71 prospective cohort studies. Br J Sports Med.

[40] Richman EL, Kenfield SA, Stampfer MJ, Paciorek A, Carroll PR, Chan JM (2011). Physical activity after diagnosis and risk of prostate cancer progression: data from the Cancer of the prostate strategic urologic research endeavor. Cancer Res.

[41] Friedenreich CM, Wang Q, Neilson HK, Kopciuk KA, McGregor SE, Courneya KS (2016). Physical activity and survival after prostate cancer. Eur Urol.

[42] Wang Y, Jacobs EJ, Gapstur SM, Maliniak ML, Gansler T, McCullough ML, Stevens VL, Patel AV (2017). Recreational physical activity in relation to prostate cancer-specific mortality among men with nonmetastatic prostate cancer. Eur Urol.

[43] Smith, L., et al., Cancer survivors' attitudes towards and knowledge of physical activity, sources of information, and barriers and facilitators of engagement: a qualitative study. Eur J Cancer Care (Engl), 2017. 26(4). 10.1111/ecc.12641 28135016

[44] Koutoukidis, D.A., et al., Attitudes, challenges and needs about diet and physical activity in endometrial cancer survivors: a qualitative study*.* Eur J Cancer Care (Engl), 2016. 10.1111/ecc.12531 27324208

[45] Fisher, A., et al., Recall of physical activity advice was associated with higher levels of physical activity in colorectal cancer patients. BMJ Open, 2015. **5**(4). 10.1136/bmjopen-2014-006853 PMC4420935 25922098

[46] Jones LW, Courneya KS, Fairey AS, Mackey JR (2004). Effects of an oncologist’s recommendation to exercise on self-reported exercise behavior in newly diagnosed breast cancer survivors: a single-blind, randomized controlled trial. Ann Behav Med.

[47] Williams K, Beeken RJ, Fisher A, Wardle J (2015). Health professionals’ provision of lifestyle advice in the oncology context in the United Kingdom. European Journal of Cancer Care.

[48] Koutoukidis DA, Lopes S, Fisher A, Williams K, Croker H, Beeken RJ (2018). Lifestyle advice to cancer survivors: a qualitative study on the perspectives of health professionals. BMJ Open.

[49] Cantwell M, Walsh D, Furlong B, Moyna N, McCaffrey N, Boran L, Smyth S, Woods C (2018). Healthcare professionals’ knowledge and practice of physical activity promotion in cancer care: challenges and solutions. Eur J Cancer Care (Engl).

[50] Murphy JL, Girot EA (2013). The importance of nutrition, diet and lifestyle advice for cancer survivors - the role of nursing staff and interprofessional workers. J Clin Nurs.

[51] van Veen MR (2017). Improving oncology Nurses’ knowledge about nutrition and physical activity for Cancer survivors. Oncol Nurs Forum.

[52] Karvinen KH, McGourty S, Parent T, Walker PR (2012). Physical activity promotion among oncology nurses. Cancer Nurs.

[53] Keogh JW, Pühringer P, Olsen A, Sargeant S, Jones LM, Climstein M (2017). Physical activity promotion, beliefs, and barriers among Australasian oncology nurses. Oncol Nurs Forum.

[54] Roberts AL, Fisher A, Smith L, Heinrich M, Potts HWW (2017). Digital health behaviour change interventions targeting physical activity and diet in cancer survivors: a systematic review and meta-analysis. J Cancer Surviv.

[55] Roberts AL, Potts HWW, Koutoukidis DA, Smith L, Fisher A (2019). Breast, prostate, and colorectal cancer survivors’ experiences of using publicly available physical activity Mobile apps: qualitative study. JMIR Mhealth Uhealth.

[56] Collado-Borrell R, Escudero-Vilaplana V, Calles A, Garcia-Martin E, Marzal-Alfaro B, Gonzalez-Haba E, Herranz-Alonso A, Sanjurjo-Saez M (2018). Oncology patient interest in the use of new technologies to manage their disease: cross-sectional survey. J Med Internet Res.

[57] Perski, O., et al., Conceptualising engagement with digital behaviour change interventions: a systematic review using principles from critical interpretive synthesis. Transl Behav Med, 2017. **7**(2). 10.1007/s13142-016-0453-1 PMC5526809 27966189

[58] O’Connor S (2016). Understanding factors affecting patient and public engagement and recruitment to digital health interventions: a systematic review of qualitative studies. BMC Med Inform Decis Mak.

[59] Craig, P., et al., *Developing and evaluating complex interventions: the new Medical Research Council guidance.* Br Med J, 2008. 337(7676). 10.1136/bmj.a1655 PMC2769032 18824488

[60] Bradbury, K., et al., Developing digital interventions*: a methodological guide.* Evidence-Based Complementary and Alternative Medicine, 2014. 10.1155/2014/561320 PMC3932254 24648848

[61] Braun V, Clarke V (2006). Using thematic analysis in psychology. Qual Res Psychol.

[62] Scott, G., Motivational interviewing 1: background, principles and application in healthcare. Nurs Times, 2010. 106(34). 20882828

[63] The Lancet Oncology, *Exercise and cancer treatment: balancing patient needs.* Lancet Oncology, 2018. **19**(6): p. 715–715. 10.1016/S1470-2045(18)30376-0 29893247

[64] Santa Mina D (2018). *Exercise as part of routine cancer care*. Lancet Oncology.

